# Knowledge and risk perception of e-cigarettes and hookah among young adults in Anambra State, Nigeria

**DOI:** 10.1186/s12889-025-23790-1

**Published:** 2025-07-25

**Authors:** Nkem Hillary Okeke, Ndubuisi Paris Obi, Chinomnso Chinanuekpere Nnebue

**Affiliations:** 1 Department of Community Medicine, Faculty of Basic Medical Sciences, Nnamdi Azikiwe University, Nnewi Campus, Anambra, Nigeria; 2 Department of Anatomy, Faculty of Basic Medical Sciences, Nnamdi Azikiwe University, Nnewi Campus, Anambra, Nigeria; 3 Developmental Neurobiology and Forensic Anatomy Unit, Department of Anatomy, University of Ibadan, Ibadan, 200212 Nigeria

**Keywords:** E-cigarettes, Hookah, Knowledge, Risk perception, Anambra State, Health hazards

## Abstract

**Background:**

E-cigarettes and hookah have gained significant popularity, particularly among young people in recent years, despite limited research on their safety and efficacy. Advertisements for these products are widespread across various media platforms, further increasing their popularity and raising awareness among young adults. This study aimed to assess the knowledge and risk perception of e-cigarettes and hookah among young adults in Anambra State, Nigeria.

**Method:**

This descriptive cross-sectional study was conducted in three tertiary institutions, selected through simple random sampling from the three Senatorial Districts of Anambra State. Participants (*n* = 272), aged 18–35 years, were recruited using a multi-stage stratified random sampling technique. A self- administered, semi- structured questionnaires was shared amongst students of the three tertiary institutions that met the inclusion criteria. Descriptive statistics were generated, and chi-square tests were conducted to determine the level of significance at *p* < 0.05.

**Result:**

The study showed that the majority of participants (66.7%) sourced their information about e-cigarettes and hookah primarily from the internet/media, although 58.5% reported never having seen these substances in person. Among the participants, 7.4% reported using both e-cigarettes and hookah, with the highest prevalence of use seen in the 25–29 age group. The first use of these substances commonly occurred in cafés/restaurants (7.4%) and nightclubs (6.6%). Majority (64.7%) perceived both e-cigarettes and hookah as equally harmful in terms of health risks, while 50.7% considered both substances equally addictive. Participants identified respiratory (65.4%) and cardiovascular (71.7%) diseases as the primary health risks associated with the use of these substances.

**Conclusion:**

This study demonstrates a high level of awareness of the health risks, addictive nature, and harmful effects of e-cigarette and hookah use among young adults in Anambra State. However, there appears to be a lack of in-depth, comprehensive understanding of the broader implications of these risks. The prevalence of use in social settings reveals the need for targeted, detailed education on their risks. Incorporating these products into smoke-free policies and regulating access in environments frequented by young adults are essential to curbing their growing use and associated health risks.

## Introduction

In recent years, the use of alternative smoking devices, such as e-cigarettes and hookah, has gained significant popularity worldwide, particularly among young adults [1–5]. Electronic nicotine delivery systems (ENDS), commonly known as e-cigarettes, vaping devices, or vape pens, are novel battery-operated devices designed to vaporize a flavored or unflavored liquid, often containing nicotine, for inhalation without combustion of tobacco [6, 7]. E-cigarettes are composed of a rechargeable battery, a heating element also known as an atomizer, and a liquid solution. This liquid generally contains a solvent, which is often a combination of propylene glycol (PG) and vegetable glycerin (VG), along with nicotine and various additives, including flavorings [8, 9].

To generate an aerosol, a person who uses e-cigarette inhales through the mouthpiece, triggering an airflow sensor that activates the atomizer. This process heats the e-liquid, producing a vapor that is then inhaled [2, 6, 9, 10].

E-cigarettes have experienced significant growth in popularity. The awareness and usage, particularly among people who smoke, have been influenced by advertisement in various media including television (TV), radio, print, online, and at retail point of sale, similar to the impact of tobacco promotions on consumer behavior [11]. E-cigarette use has been on the rise among middle and high school students in the United States. According to the National Youth Tobacco Survey, the percentage of students in grades 6–12 who had ever tried e-cigarettes increased from 3.3 to 6.8%, while current usage rose from 1.1 to 2.1% between 2011 and 2012 [12]. This trend is not limited to Western nations; in Nigeria, and particularly in Anambra State, these devices have become more accessible and are gaining traction among young people. E-cigarettes and hookah are often marketed as safer alternatives to traditional cigarettes [2], but emerging evidence suggests that their health risks, particularly among young and impressionable populations, may be significant [13, 14].

Young people and teenagers are especially drawn to e-cigarettes for a variety of reasons, such as curiosity, peer pressure, social standing, the abundance of delicious flavors and the fact that e-cigarettes are readily available at malls and convenience stores [15]. This increasing prevalence of e-cigarette use among adolescents raises concerns about the possibility that e-cigarettes could lead to nicotine dependence or act as a gateway to traditional tobacco cigarettes, although it is still hotly contested [14, 16]. The higher incidence of mental health conditions like depression is another significant negative effect connected with e-cigarettes use. E-cigarettes are associated with anxiety disorders, which are characterized by excessive worry, fear, or anxiety that interferes with day-to-day functioning [17]. The use of e-cigarettes is reported to be more common among individuals with depression or anxiety compared to those without mental health conditions [18]. In fact, smoking rates among people with mental health conditions are estimated to be about 70% higher than in those without such conditions [18].

Furthermore, there is evidence from international studies demonstrating a concerning connection between e-cigarette use and mental health issues. According to the study carried out on American college students, it showed that e-cigarette use was associated with depressive symptoms and other mental health problems. It was found that students experiencing depression are more likely to use e-cigarettes than their peers who are not depressed [19]. However, in another study conducted in 2017 it was shown that the association between e-cigarette use and depression in adolescents was minimal [20]. These conflicting findings show the need for further research, particularly in different cultural and geographic contexts. Despite the increasing popularity of these devices, there is a paucity of data on young people’s knowledge and risk perception associated with the use of e-cigarettes and hookah in Nigeria [21]. Therefore, this study sought to explore the knowledge and risk perception of e-cigarettes and hookah among youths in Anambra State, Nigeria, to better understand the potential health implications associated with their use.

## Method

### Study design

This study employed a descriptive cross-sectional design to assess the knowledge and risk perception of e-cigarettes and hookah among young adults in Anambra State, Nigeria, specifically targeting students from Nnamdi Azikiwe University Awka, Nwafor Orizu College of Education Nsugbe, and the College of Health Sciences, Nnamdi Azikiwe University Nnewi Campus.

### Study population

Participants (*n* = 272) aged 18–35 years were recruited from three higher institutions across the three senatorial districts of Anambra State: Anambra Central, Anambra South, and Anambra North. The study targeted students who had been enrolled in their respective institutions for at least one year. The study targeted students who had been enrolled in their respective institutions for at least one year, including both undergraduate and postgraduate students.

### Inclusion criteria

1. Young adults admitted in the selected Higher Institutions for at least one year.
2. Full-time students who were officially enrolled during the study period.

### Exclusion criteria

1. Those selected as above but were not present during the study.
2. Those who were present but declined assent or full informed consent.

### Sample size determination

The sample size was calculated using the Cochran formula [22] { n = Z^2^ x P x (1-p)/d^2^ }, with a 95% confidence interval (Z = 1.96), 50% prevalence (*P* = 0.5), and 5% margin of error (d = 0.05), yielding a sample size of 384. After applying the finite population correction formula for a population of 500, the sample size was adjusted to 217. Considering a 10% non-response rate, the sample size was further increased to 241, and then rounded up to 272 (the total population size used for this study) to enhance study power, accounting for potential non-response and improperly filled questionnaires.

### Sampling technique

A multistage sampling technique was employed.

- Stage 1: Selection of Senatorial Zones: The study was conducted across the three Senatorial Districts of Anambra State.
- Stage 2: Selection of Higher Institutions: One institution was selected from each Senatorial Zone using simple random sampling through balloting. In the balloting method, names of all eligible institutions in each district were written on separate slips of paper, placed in a container, shuffled, and one slip was randomly drawn without replacement to determine the selected institution.
- Stage 3: Selection of Participants: Students aged 18 to 35 from the selected institutions were randomly chosen.
- Stage 4: A total of 272 participants were recruited using a simple random sampling technique.

### Data collection

Data were collected using a semi-structured, self-administered questionnaire adapted from a UK database survey which was developed according to the objectives of this study. The questionnaire comprised four sections (A-D). Section A contained socio-demographic data of respondents, while sections B, C, and D assessed knowledge and risk perception of e-cigarettes and hookah. Awareness was assessed by asking participants if they had ever heard of e-cigarettes or hookah, with response options of “Yes” or “No.” Participants were assured of confidentiality, and informed consent was obtained prior to administering the questionnaires.

### Statistical analysis

The collected data were analyzed using the Statistical Package for the Social Sciences (SPSS) version 25. Descriptive statistics were generated, and chi-square tests were conducted to determine the level of significance at *p* < 0.05.

## Results

Table 1 presents summary of the socio-demographic profiles of the participants. From the table, it can be observed that a total of 272 participants (52.9% males) participated in the study, with a mean age of 23.19 ± 3.46 years. The majority of participants were single (94.1%), predominantly belonged to the Catholic denomination (47.8%), and identified as members of the Igbo tribe (87.1%). Over half of the participants (55.5%) attained post-secondary (tertiary) education, with 33.8% holding bachelor’s qualifications and 1.5% possessing a Master’s degree (MSc), indicating that postgraduate students were also represented in the study population.

**Table 1:** Socio-demographic profiles of the participants

Variables	Class	Frequency	Percentage
Gender	Gender	144	52.9
	Female	128	47.1
Marital status	Single	256	94.1
	Married	15	5.5
	Other	1	0.4
Religion	Catholic	130	47.8
	Anglican	54	19.9
	Pentecostal	71	26.1
	Muslim	4	1.5
	Other	13	4.8
Tribe	Igbo	237	87.1
	Hausa	17	6.3
	Yoruba	5	1.8
	Other	13	4.8
Qualification	NCE	32	11.8
	OND	18	6.6
	HND	5	1.8
	BSc	92	33.8
	MSc	4	1.5
	Other	121	44.5

Table 2 shows summary of the descriptive statistics of the knowledge and awareness of E-Cigarettes and Hookah Among Participants. Most (65.4%) of the participants reported to have heard of e-cigarettes and hookah with internet/media (66.7%) being the most frequent source of information. Despite this, a majority (58.5%) had not seen either e-cigarettes or hookah. When identifying health hazards, cardiovascular (71.7%) and respiratory (65.4%) issues were most frequently recognized as significant risks. Furthermore, the majority (64.7%) perceived both e-cigarettes and hookah as equally harmful in terms of health risks, while 50.7% considered both substances equally addictive.

**Table 2:** Knowledge about e-cigarettes and hookah among the participants

Knowledge Questions	Response options	Frequency	Percentage
If yes, how did you get to hear about it?	Internet / media	126	66.7
	School	37	19.6
	Books	27	14.3
	Friends	105	55.6
	Other	−	1.6
Which of them have you seen?	None	159	58.5
	Both	82	30.1
	Other	31	11.4
Which among these do you identify as important health hazards of Hookah and e-cigarettes smoking?	Respiratory problems	178	65.4
	Cardiovascular impairments	195	71.7
	Hematologic Impairments	122	44.9
	Pregnancy Hazards	62	22.8
	Diarrhea	99	36.4
Which among E-CIGARETTE and Shisha/Hookah are more addictive according to your perception?	Shisha/Hookah (water-pipe) smoking is more addictive	51	18.8
	e-Cigarette smoking is more addictive	51	18.8
	Both are equally addictive	138	50.7
Where do you rate the health hazards of habitual hookah smoking in comparison to those of E-cigarette smoking?	Cigarette smoking is more harmful	43	15.8
	Water-pipe smoking is more harmful	28	10.3
	Both are equal in terms of health risks	176	64.7

NB: To protect participant confidentiality, cell frequencies less than 4 have been replaced with "–". However, percentages are reported regardless of cell size

Table 3 shows the statistics of the usage of e-cigarettes and hookah among participants. Out of the participants, 6 (2.2%) reported using both substances, 2 (0.7%) were using only shisha, and 4 (1.5%) were using only hookah at the time of data collection. Among the people who use e-cigarettes and hookah currently, most people who use hookah (75.5%) smoked less than once a year. The age group with the highest proportion of people who smoked hookah for the first time was 25–29 years, accounting for 7.0% of participants. A combined 9.2% of the participants were considering using any of traditional cigarette, smokeless tobacco, hookah, electronic cigarette or their combinations in the future.

**Table 3:** Usage of e-cigarettes and hookah among the participants

Usage Questions	Response options	Frequency	Percentage
If you are currently using any, specify	Both	6	2.2
	Shisha	−	0.7
	Hookah	4	1.5
Which one of the following did you use first?	Traditional cigarettes	23	8.5
	Smokeless tobacco (snus or chewing tobacco)	8	2.9
	Hookah (Water pipe tobacco smoking or Shisha)	17	6.3
	Electronic cigarettes	–	1.1
	None of these	221	81.3
Which of the following are you currently using?	Traditional cigarettes	–	0.7
	Smokeless tobacco (snus or chewing tobacco)	–	1.1
	Hookah (Water pipe tobacco smoking or Shisha)	9	3.3
	Electronic cigarettes	–	0.4
	None of these	256	94.1
	All of the above	–	0.4
Which are you likely to use in the future?	Traditional cigarettes	5	1.8
	Smokeless tobacco (snus or chewing tobacco)	4	1.5
	Hookah (Water pipe tobacco smoking or Shisha)	6	2.2
	Electronic cigarettes	7	2.6
	None of these	248	90.8
	All of the above	–	1.1
Please explain why to the answer in the future usage?	Religion	90	33.1
	Health	97	35.7
	Personal	85	31.3
	Pleasure	–	0.0
Have you ever used a hookah to smoke tobacco (even one or two puffs)?	No	229	84.2
	Yes	43	15.8
Which of the following choices best describes how often you smoke tobacco using a hookah?	Less than once a year	17	6.3
	At least once a year, but not monthly	23	8.5
	At least once a month, but not weekly	8	2.9
	At least once a week, but not daily	–	1.1
	At least once a day, or most days each month	–	1.1
How old were you when you FIRST used a hookah?	Younger than 9 years old	–	1.1
	10-12 years old	–	0.7
	13-15 years old	5	1.8
	16-18 years old	7	2.6
	19-21 years old	14	5.1
	22-24 years old	0	0.0
	25-29 years old	19	7.0
	30 years old or older	4	1.5

NB: To protect participant confidentiality, cell frequencies less than 4 have been replaced with "–". However, percentages are reported regardless of cell size

Table 4 shows the summary of the statistics of the usage of e-cigarettes and hookah among relatives, friends, and significant others of participants.

**Table 4:** Usage of e-cigarettes and hookah among relatives, friends and significant orders of the participants

Questions	Response options	Frequency	Percentage
Do one or more of your close friends use electronic cigarettes or Hookah?	Yes	90	33.1
	No	97	35.7
	Don’t know	85	31.3
Does your mother or father (guardian) use electronic cigarettes or hookah?	Yes both my mom and dad use	_	0.7
	Yes, my mom uses	_	0.4
	Yes, my dad uses	4	1.5
	No, neither my mom nor dad use	265	97.4
Who else in your family use electronic cigarettes or hookah?	Brother (s)/Sister(s)	24	8.8
	Grandmother	4	1.5
	Grandfather	0	0.0
	Uncle	9	3.3
	Aunt	0	0.0
	Other, cousin	10	3.7
	No one uses	0	0.0
	I don’t know	139	51.1
	8	82	30.1
	9	4	1.5
Who were you with when you FIRST used a hookah?	No one, I was alone	8	2.9
	With one friend	21	7.7
	With more than one friend	17	6.3
	With a family member	5	1.8
	With more than one family member	0	0.0
	With a new acquaintance	_	0.4
Where were you when you FIRST used hookah?	In a cafe or restaurant	20	7.4
	In my own home (apartment, condo, house)	_	0.4
	In the night club	1.8	6.6
	At a family member's home	4	1.5
	At a fraternity/sorority house	_	1.1
	At a friend's or acquaintance's home	5	1.8

NB: To protect participant confidentiality, cell frequencies less than 4 have been replaced with "–". However, percentages are reported regardless of cell size

Approximately one-third of participants reported having one or more close friends (2.6%) and one or both parents (8.8%) who used hookah or e-cigarettes. The majority of the people who use e-cigarettes and hookah indicated they were with friends during their first experience with hookah. The most common locations for first use were cafés/restaurants (7.4%) and nightclubs (6.6%).

Table 5 shows the association of awareness of E-Cigarettes and Hookah with Selected Socio-Demographic Variables. No significant associations were found between awareness of e-cigarettes and hookah and gender, marital status, religion, tribe, or educational qualifications (*p* > 0.05).

**Table 5:** Showing the association between awareness of e-cigarette and hookah and selected socio-demographic variables of the participants

Variable	Class	Awareness (n(%))			X^2^	P
		Yes	No	Maybe		
Gender	Male	98(68.1)	38(26.4)	8(5.6)	1.11	0.58
	Female	80(62.5)	38(29.7)	10(7.8)		
Marital status	Single	165(64.5)	75(29.3)	16(6.3)	4.72	0.32
	Married	12(80.0)	– (6.7)	– (13.3)		
	Others	– (100.0)	0(0.0)	0(0.0)		
Religion	Catholic	83(63.8)	41(31.5)	6(4.6)	15.07	0.06
	Anglican	41(75.9)	9(16.7)	4(5.6)		
	Pentecostal	45(63.4)	22(31.0)	4(5.6)		
	Muslim	–(25.0)	– (50.0)	– (25.0)		
	Others	8(61.5)	– (15.4)	– (23.1)		
Tribe	Igbo	151(63.7)	70(29.5)	16(6.8)	8.10	0.23
	Housa	15(88.2)	– (5.9)	– (5.9)		
	Yoruba	5(100.0)	0(0.0)	0(0.0)		
	Others	7(53.8)	5(38.5)	– (7.7)		
Qualifications	NCE	23(71.9)	7(21.9)	– (6.3)	7.62	0.67
	OND	13(72.2)	3(16.7)	– (11.1)		
	HND	4(80.0)	– (20.0)	0(0.0)		
	BSc	60(65.2)	23(25.0)	9(9.8)		
	MSc	– (75.0)	– (25.0)	0(0.0)		
	Others	75(62.0)	41(33.9)	5(4.1)		

NB: To protect participant confidentiality, cell frequencies less than 4 have been replaced with "–". However, percentages are reported regardless of cell size

*NCE* Nigerian Certificate in Education, *OND* Ordinary National Diploma, *HND* Higher National Diploma, *BSc* Bachelor of Science, *MSc* Master of Science

Table 6 shows the association of usage of E-Cigarettes and Hookah with Selected Socio-Demographic Variables. A significant association was noted between gender and usage, with a higher proportion of males having used the substances compared to females (χ² = 17.04, *p* < 0.01). Additionally, tribe significantly influenced usage, with a greater proportion of Hausas using the substances compared to other tribes (χ² = 89.00, *p* < 0.01). Educational qualifications were also significantly associated with usage, showing that participants with higher educational attainment tended to indulge less in the use of e-cigarettes and hookah (χ² = 67.12, *p* < 0.01).

**Table 6:** Showing the association between Usage of e-cigarette and hookah and selected socio-demographic variables of the participants

Variable	Class	Usage (n(%))			X^2^	*P*
		None	Both	Other		
Gender	Male	109(75.7)	27(18.8)	8(5.6)	17.04	<0.01*
	Female	120(93.8)	5(3.9)	– (2.3		
Marital status	Single	215(84.0)	30(11.7)	11(4.3)	0.88	0.32
	Married	13(86.7)	– (13.3)	0(0.0)		
	Others	– (100.0)	0(0.0)	0(0.0)		
Religion	Catholic	106(81.5)	19(14.6)	5(3.8)	11.09	0.20
	Anglican	43(79.6)	8(14.8)	– (5.6)		
	Pentecostal	64(90.1)	5(7.0)	– (2.8)		
	Muslim	– (75.0)	0(0.0)	– (25.0)		
	Others	13(100.0)	0(0.0)	0(0.0)		
Tribe	Igbo	210(88.6)	17(7.2)	10(4.2)	89.00	<0.01*
	Housa	– (17.6)	14(82.4)	0(0.0)		
	Yoruba	4(80.0)	– (20.0)	0(0.0)		
	Others	12(92.3)	0(0.0)	– (7.7)		
Qualifications	NCE	15(46.9)	17(53.1)	0(0.0)	67.13	<0.01*
	OND	14(77.8)	4(22.2)	0(0.0)		
	HND	5(100.0)	0(0.0)	0(0.0)		
	BSc	82(89.1)	6(6.5)	4(4.3)		
	MSc	4(100.0)	0(0.0)	0(0.0)		
	Others	109(90.1)	5(4.1)	7(5.8)		

NB: To protect participant confidentiality, cell frequencies less than 4 have been replaced with "–". However, percentages are reported regardless of cell size

KEY: *Significant at *p*<0.05

## Discussion

This study is among the few to quantitatively examine the knowledge and risk perceptions of e-cigarette and hookah use among young people in Nigeria. The present research aimed to assess the understanding and perceptions of risks associated with e-cigarettes and hookah among young adults in Anambra State, Nigeria. This is particularly relevant given the alarming global rise in the use of these substances, which pose significant yet preventable health risks. As e-cigarettes and hookah become increasingly popular among young populations, it is crucial to explore how awareness and risk perceptions shape their usage patterns [23]. Understanding these dynamics is essential for informing public health interventions aimed at reducing tobacco and nicotine consumption among vulnerable young adults’ demographics. The young adults were targeted in this study (as revealed by the mean age = 23.19 ± 3.46 years) because they have been reported as the group most liable to indulge in the usage of these substances, with more than 70% of cigarette and hookah smoking starting during adolescence [24]. Majority of the participants were Igbo’s and Christians who were mainly Catholics and Anglicans. This is not surprising as Igbos are the natives of the setting of this study, and are mainly Christians of Catholic and Anglican denominations. Educational attainment of the participants was high as majority attained tertiary education. This is in line with previous reports that Igbos are one of the most educated tribes in Nigeria [25].

Most of the participants reported to have heard of e-cigarettes and hookah with internet/media being the most frequent source of information. This is understandable, considering the fact that usage of internet and social media has unprecedentedly increased in recent times, and act as a medium for fast dissemination of information [26]. However, over half of the participants had not seen any of e-cigarette and hookah suggesting that their awareness did not come from having direct contacts with the substances. The participants of the present study seemed to be knowledgeable about the health hazards (especially respiratory and cardiovascular) of e-cigarette and hookah. This might not be unconnected to their enlightenment from media or internet. Cigarette smoking has been reported to associate with development of several systemic conditions (including cancer, cardiovascular and pulmonary diseases), oral diseases (including oral cancer, periodontal diseases and peri-implantitis) and so on [27]. People who use hookah have actually been reported to be exposed to many of the same toxic compounds/by-products as conventional cigarette users, often at higher levels, which might lead to more severe negative health effects [28]. Majority of the participants in the present study rated both e-cigarette and hookah as having equal addictive tendencies. The two substances have been reported to deliver the dependence-producing drug nicotine [27]. A total of 7.4% of the participants in the present study reported to have used e-cigarette and hookah while 3.7% were still using them at the point of data collection. Previous reports [2, 5, 24], have revealed that the use of e-cigarette and hookah had been an emerging trend especially among the young adults. However, the recorded point prevalence (3.7%) of hookah use in the current study falls below 9–12% recorded in Syria [29], 30% in Jordan [30], 9.2% and 25.5% in Iran [24], 29% in Canada [31], 12–15% in the US, 12% in Estonia, 8% in the UK, 25% in Lebanon, 15% in Syria and 33% in Pakistan [29]. This may be a pointer to the fact that hookah and e-cigarette use is not yet as rife in Nigeria as in many other countries. This is further buttressed by the fact that majority of the people who smoked hookah in this study indulged in the act only once a year unlike more frequent usage in other climes [24]. People who use these substances were spread across different age groups between 10 and 30 years. Furthermore, a combined 4.8% prevalence of the participants grandparents, aunts and uncles were reported to be using hookah unlike a combined higher 12.5% prevalence of siblings and cousins (who are understandably younger than the previous group) of the participants that were using hookah. This may be supporting the previous reports that hookah usage is more popular among youth [30].

A combined 9.2% of the participants were considering using any of traditional cigarette, smokeless tobacco, hookah, electronic cigarette or their combinations in the future. This is similar to 7.9% pooled probabilities of cigarette initiation in people who never used e-cigarette reported in a systematic review [22], and may be a pointer to the increasing popularity of e-cigarette usage among young adults as reported in literature [27]. This may be a cause for concern as e-cigarette smoking initiation had been reported to significantly associate with subsequent cigarette usage [22]. This might have also highlighted the need for strong e-cigarette regulation in order to curb young adults access to e-cigarette and hookah which are still easily accessible [22]. This accessibility has probably contributed in making e-cigarette and hookah most popular form of tobacco smoking, overtaking the conventional cigarette in recent years [27]. This increase in usage of hookah has also been partly attributed to misperception of hookah being less harmful than cigarettes and the availability of different but appealing flavors [32].

The proportion of participants (33.1%) that reported having one/more close friends smoking e-cigarette and hookah is almost double the proportion (18.9%) of thos that reported having a relative (parents, grandparents, siblings, cousins, aunts and uncles) smoking the substances. This may be highlighting the influence peer pressure may be having on initiation of e-cigarette and hookah smoking, as many participants would have been likelier to be persuaded to indulge in the act by their friends than by a relative. This assumption is supported by the fact that majority of the people who used it reported being with their friend(s) when they first used hookah. Peer influence has been reported as a major factor in hookah smoking [23]. Café/restaurant and night club were the most frequent locations where people first used hookah in the present study. These substances have been reported to be commonly used in public places including social parties and cafés [23]. Therefore, this may serve as a wake-up call for the government to implement stricter smoke-free policies in public spaces and raise awareness on the dangers of passive smoking.

There was significant association of gender with usage of e-cigarette and hookah among the participants of this study with more males having used the substances than their female counterparts. This is contrary to previous Iranian study [24] where more female high school students were smoking cigarette and hookah than their male counterparts. The fact that Igbo’s have a patriarchal culture where women are expected to be less adventurous than men might have discouraged Igbo female young adults from indulging in hookah and e-cigarette smoking like the male. There was also a significant association between usage of e-cigarette and hookah and tribe with more proportion of Hausas having significantly used the substances than other tribes. Varied prevalence of e-cigarette and hookah smoking had been reported across different cultures and geographical locations with higher prevalence reported among Middle Eastern countries who are mainly Muslims [29]. The fact that Hausa is a predominantly Muslim tribe who has imbibed the Arabic (Middle Eastern) cultures might have explained their higher usage of hookah and e-cigarette in this study. Qualifications of the participants also significantly associated with their usage of e-cigarette and hookah with those who had higher education attainment seeming to indulge less in the usage. This may be easily justified. More educated people might have been more enlightened on the health hazards of e-cigarette and hookah smoking than their less educated peers. This might have discouraged them from indulging in the usage of these substances.

### Strengths, weaknesses and limitations

The main strengths of the present study were to the best of our knowledge, it is the first of its kind to investigate the knowledge and risk perception of e-cigarettes and hookah among young adults in Southeastern Nigeria, addressing an important gap in public health research within the region. By employing a well-structured questionnaire and a multistage sampling technique, the study ensured robust data collection and representation across diverse participant demographics in Anambra State. Additionally, the findings provide valuable insights into sociodemographic factors, such as gender, education level, and cultural influences, that shape the usage patterns and perceptions of e-cigarettes and hookah among young adults.

Despite these strengths, the study has some limitations. The reliance on self-reported data may introduce social desirability bias, potentially leading to underreporting or overreporting of behaviors and perceptions. The cross-sectional design restricts the ability to establish causal relationships between knowledge, perceptions, and usage patterns. Furthermore, the study was conducted in a single state, which may limit the generalizability of the findings to other regions with varying cultural or sociodemographic characteristics. Finally, while the questionnaire was effective in assessing knowledge and perception, it may not have fully captured the nuanced motivations or psychological factors influencing e-cigarette and hookah use.

### Recommendations for further research

Future research should consider using a longitudinal design to explore trends and causal relationships over time. Expanding the geographic scope of the study to include other states or regions in Nigeria, with an emphasis on urban-rural comparisons, would enhance the generalizability of findings. Additionally, incorporating qualitative methods, such as focus group discussions or in-depth interviews, could provide deeper insights into the motivations, attitudes, and beliefs underlying young adults’ behavior toward e-cigarettes and hookah.

## Conclusions

This study provides valuable insights into the knowledge and risk perception of e-cigarettes and hookah among young adults in Anambra State, Nigeria. The findings reveal that a substantial majority of participants were aware of these substances, with the internet and media identified as the primary sources of information. However, despite this awareness, many participants may underestimate the associated risks, potentially leading to addiction. The demographic of people who used e-cigarettes and hookah predominantly included individuals aged 25–29 years. Notably, gender and educational qualifications were significantly associated with the usage of these substances; males were more likely to use e-cigarettes and hookah than females, while those with higher educational attainment tended to use them less frequently. These findings enrich our understanding of e-cigarette and hookah usage patterns among Nigerian young adults and can inform public health interventions and awareness campaigns targeting smoking-related behaviors. They emphasize the urgent need for enhanced awareness campaigns about the adverse health effects of e-cigarettes and hookah to promote prevention and cessation. Additionally, these insights may act as a catalyst for the Nigerian government to reinforce tobacco control regulations, addressing not only cigarette smoking but also all forms of tobacco and nicotine use.

## Data Availability

The data presented in this study are available on request from the first author (nkem.okeke@uniniger.edu.ng).
